# Supplementary material for: Three novel bird strike likelihood modelling techniques: The case of Brisbane Airport, Australia
Source: PLoS One. 2022 Dec 8;17(12):e0277794. doi: 10.1371/journal.pone.0277794 (PMC9731475; doi:10.1371/journal.pone.0277794)
Supplement: S1 Table — Dates of strike occurrences are shown together with the number of individuals involved in the occurrence. (PDF) [file pone.0277794.s002.pdf]

Species	Date (Number Struck)			
Cattle Egret	27/5/2017 (1)	17/11/2017(1)	28/11/2017 (1)	4/12/2017 (1)
	19/12/2017 (1)	14/2/2018 (1)	18/11/2018 (2)	19/11/2018 (1)
	21/12/2018 (1)			
Straw-necked Ibis	28/1/2018 (1)	22/5/2018 (1)	29/12/2018 (1)	
Nankeen Kestrel	8/5/2017 (1)	15/6/2017 (1)	7/8/2017 (1)	14/9/2017 (1)
	9/2/2018 (1)	15/3/2018 (1)	12/4/2018 (1)	24/4/2018 (1)
	5/5/2018 (1)	24/5/2018 (1)	6/7/2018 (1)	1/7/2018 (1)
	20/9/2018 (1)	24/10/2018 (1)	10/12/2018 (1)	15/1/ 2018 (1)
	6/2/2018 (1)	18/2/2019 (1)	5/3/2019 (1)	11/5/2019 (1)

Table S1. Summary of the strike information for the three hazard species involved in the study during the period of the study. Dates of strike occurrences are shown together with the number of individuals involved in the occurrence.
