# Supplementary material for: Three novel bird strike likelihood modelling techniques: The case of Brisbane Airport, Australia
Source: PLoS One. 2022 Dec 8;17(12):e0277794. doi: 10.1371/journal.pone.0277794 (PMC9731475; doi:10.1371/journal.pone.0277794)
Supplement: S2 Table — (PDF) [file pone.0277794.s003.pdf]

Variable	Cattle Egret		Straw-necked Ibis		Nankeen Kestrel	
	PC1	PC2	PC1	PC2	PC1	PC2
DayNumber	0.2737	0.04047	0.1061	11.9498	0.0035	0.0445
SolarDayNumber	0.01977	0.8946	0.8311	17.1822	0.3970	0.0578
DayofYearSin	0.0511	0.9209	1.0438	20.4983	0.6655	0.0199
DayofYearCos	5.3703	1.9489	0.4850	0.4449	2.8332	0.0243
Prev5DaysRain	0.0347	5.4551	0.7711	1.2036	0.0370	0.1369
Rain	0.0677	2.515	0.1919	0.7061	0.0001	0.2553
Temperature	2.4499	2.9569	0.9444	13.2867	1.0432	0.0103
AirfieldHarassments	7.3477	6.4636	9.7532	0.9710	10.0685	4.5549
AllCount	7.2849	8.5513	6.4996	0.0010	5.8611	1.2949
AllHarassed	6.8451	7.0138	10.5973	0.5523	10.2476	2.4255
AllObserved	9.9517	0.5974	11.8126	0.3390	12.3181	1.1856
AllHarassments	7.6388	0.2259	5.0160	4.9388	11.1806	1.0521
X1_Count	5.7193	0.1602	3.8539	1.6558	3.2154	2.1346
X1_Harassed	6.9253	7.4439	9.4121	1.3142	8.9372	7.5284
X1_Observed	8.3464	4.8897	9.6902	1.6451	10.4593	1.8035
X1_SpeciesHarassments	7.1989	6.4639	8.9079	1.8418	9.0991	7.6441
X1_Harassments	0.5459	4.7389	4.9740	1.2304	1.6704	2.2352
X2_Count	2.9974	0.1402	1.4131	0.0030	1.4046	6.7431
X2_Observed	3.3326	0.0033	3.1604	0.0134	1.8823	4.0738
X2_Harassments	0.0091	0.2178	0.8889	0.2872	0.2826	0.0071
X5_Count	4.8532	2.8915	2.2570	6.3327	2.6238	2.8701
X5_Observed	5.1269	2.4792	3.9609	5.8846	2.7591	2.3609
X5_Harassments	0.0102	0.5947	1.2529	1.6662	0.1146	0.1173
X8_Count	2.2735	0.1438	0.0097	0.4730	0.2654	1.1682
X8_Observed	2.2839	0.0020	0.2612	0.7091	0.2223	1.0375
X10_Count	1.4785	16.3509	0.9464	2.4325	1.1601	4.7127
X10_Observed	1.4894	15.8946	0.9507	2.4141	1.2165	4.3889
X10_Harassments	0.0745	0.0009	0.0086	0.0235	0.0321	0.1125

Table S2. Complete listing of the two principal components, including variable loadings, responsible for the greatest variation as used in Clustering models.
