# Supplementary material for: Three novel bird strike likelihood modelling techniques: The case of Brisbane Airport, Australia
Source: PLoS One. 2022 Dec 8;17(12):e0277794. doi: 10.1371/journal.pone.0277794 (PMC9731475; doi:10.1371/journal.pone.0277794)
Supplement: S3 Table — (PDF) [file pone.0277794.s004.pdf]

Parameters	Cattle Egret	Straw-necked Ibis	Nankeen Kestrel
Solar day 0	1-May	1-May	1-Nov
Gaussian		Height $a_1 = 0.48$<br>Centre $b_1 = 1$<br>Width $c_1 = 0.4$	Height $a_1 = 8.5$<br>Centre $b_1 = -1$<br>Width $c_1 = 0.3$
		Height $a_2 = 0.48$<br>Centre $b_2 = -1.1$<br>Width $c_2 = 0.4$	
Sigmoid	Height $a_1 = 58$<br>Centre $b_1 = 0$<br>Width $c_1 = 0.5154$<br>Steepness $k_1 = 11.2$		Height $a_2 = 1.9$<br>Centre $b_2 = 0.55$<br>Width $c_2 = 0.9$<br>Steepness $k_2 = 15$

Table S3. Configuration parameters for the gaussian and sigmoid functions used in Algebraic models for each of the hazard species, and the 'day 0' value for each of the hazard species.
