# Supplementary material for: Three novel bird strike likelihood modelling techniques: The case of Brisbane Airport, Australia
Source: PLoS One. 2022 Dec 8;17(12):e0277794. doi: 10.1371/journal.pone.0277794 (PMC9731475; doi:10.1371/journal.pone.0277794)
Supplement: Attachment — Submitted filename: 20220708 Response to Reviewers Letter.pdf [file pone.0277794.s005.pdf]

Response to Reviewers

Due: Jul 08, 2022

Ref: PONE-D-21-25171 "The ABC of Bird Strikes at BAC: Modelling bird strike likelihood at Brisbane Airport".

Comment #	Comment	Author Team Response
**Editor's Comments**		
E.2	We note that one or more of the authors have an affiliation to the commercial funders of this research study : Brisbane Airport Corporation	This is correct. We have amended the Funding Statement to reflect this (see response to E.2.a.1).
E.2.a.1	Please provide an amended Funding Statement declaring this commercial affiliation, as well as a statement regarding the Role of Funders in your study. If the funding organization did not play a role in the study design, data collection and analysis, decision to publish, or preparation of the manuscript and only provided financial support in the form of authors' salaries and/or research materials, please review your statements relating to the author contributions, and ensure you have specifically and accurately indicated the role(s) that these authors had in your study. You can update author roles in the Author Contributions section of the online submission form.	Authors receiving funding: AtH, MW, RA, BB, BC, JR<br>Grant: Direct funding<br>Funder: Brisbane Airport Corporation<br>Website: https://www.bne.com.au/corporate<br><br>*Funding was awarded to the following QUT CIs: AtH, MW, RA. The grant funding was used for (partial) salaries of the following authors (as QUT employees): BB, BC, RA. The funder provided support in the form of salaries for author JR (as an employee of BAC). The specific roles of these authors are articulated in the 'author contributions' section. The funder did not have any role in the analysis, or preparation of manuscript. The funder was involved in validating the study design, and made historical data collected by its Wildlife Hazard Management team available to the researchers. The funder was also involved in the decision to publish.*
E.2.a.2	Please also include the following statement within your amended Funding Statement.<br><br>"The funder provided support in the form of salaries for authors [insert relevant initials], but did not have any additional role in the study design, data collection and analysis, decision to publish, or preparation of the manuscript. The specific roles of these authors are articulated in the 'author contributions' section."<br><br>If your commercial affiliation did play a role in your study, please state and explain this role within your updated Funding Statement.	See E.2.a.1 for revised Funding Statement.
E.2.b.1	Please also provide an updated Competing Interests Statement declaring this commercial affiliation along with any other relevant declarations relating to employment, consultancy, patents, products in development, or marketed products, etc.	Updated Competing Interest statement:<br><br>*I have read the journal's policy and the authors of this manuscript have the following competing interests: JR was, at the time of writing, an employee of Brisbane Airport Corporation. This does not alter our*

		adherence to PLOS ONE policies on sharing data and materials.
E.2.b.2	Within your Competing Interests Statement, please confirm that this commercial affiliation does not alter your adherence to all PLOS ONE policies on sharing data and materials by including the following statement: "This does not alter our adherence to PLOS ONE policies on sharing data and materials." (as detailed online in our guide for authors http://journals.plos.org/plosone/s/competing-interests). If this adherence statement is not accurate and there are restrictions on sharing of data and/or materials, please state these. Please note that we cannot proceed with consideration of your article until this information has been declared.	See E.2.b.1 for revised Competing Interests statement.
E.2.b.3	Please include both an updated Funding Statement and Competing Interests Statement in your cover letter. We will change the online submission form on your behalf.	Statements have been included in revised Cover Letter as requested.
E.3	Please upload a new copy of Figure 7 as the detail is not clear.	Figure has been reworked and a fresh copy uploaded.
E.4	Please include captions for your Supporting Information files at the end of your manuscript, and update any in-text citations to match accordingly.	The Supporting Information section has been updated to include more explanatory captions.<br><br>In-text citations have been updated to clearly refer to the relevant Supporting Information tables.

**Comment #**	**Comment**	**Author Team Response**
**Reviewer 1 Comments**		
Introduction		
R1.1	The Introduction nowhere acknowledges that bird strike can be a problem for bird populations as well, given the scale of incidents and mortality	We have added the following to the Introduction:<br><br>*On the other side of the coin, civil aviation, and airport operations have significantly affected bird populations. There has been an increasing number of bird strikes, generally fatal to individual birds involved, reported worldwide (annual average of 12,219 reported strikes between 2008-2015 being nearly double the annual average of 6,702 strikes reported 2001-2007) [1]. Airport operations including construction of airport infrastructure, frequent take-offs and landings, airport noise and lights, and wildlife hazard management practices aimed at reducing risk of birdstrike, e.g., spraying to remove weeds and invertebrates, drainage, and even direct killing of individual hazard species, may result in habitat fragmentation,*

		population decline, rare bird extinction adjacent to airports [2–4].
R1.2	L 51-70 contains details that are very specific to the case and not relevant for general readers. This section can be substantially revised and reduced keeping in mind the general readership.	This has been revised as:<br><br>*Certification or accreditation of an airport with the regulatory authority is contingent on the airport implementing a Wildlife Hazard Management Plan (WHMP) for the management of wildlife hazards. In Australia, compliance with the Civil Aviation Safety Authority (CASA's) Advisory Circular AC 139-26(0) Wildlife Hazard Management at Aerodromes [11] requires of airport operators, that when assessing wildlife hazard risk, "individual species should be identified and prioritised in order of risk" [11, Section 6.8]. AC 139-26(0) also points out the airport operator's requirement to provide advice to airmen at varying levels of immediacy including (i) standing caution for a bird or animal hazard that poses a constant risk, (ii) periodic , when there is a significantly increased risk, for a relatively constrained period of time, posed by a hazard species, and (iii) immediate advice of risk from the control tower to warn approaching and departing aircraft of, say, birds (or bats), detected in the flight path.*
R1.3	L 159-184 details of three hazard bird species can be reduced by retaining only the most relevant information.	This has been reduced as:<br><br>• *Nankeen Kestrels are small, non-migratory raptors (bird of prey) which exhibit a strong seasonal pattern in their mode of hunting [18], alternating between hover-hunting (riding thermals, particularly over concrete runways and taxiways where aircraft are moving) and still-hunting (launching from a perch). Nankeen Kestrels are the most frequently struck bird species at Brisbane airport, with strikes throughout the year.*<br>• *Cattle Egrets are migratory and have high numbers from November till February (southern summer) with markedly fewer individuals observed in March–October (southern winter).*<br>• *The Straw-necked Ibis population on/around the airport is the most volatile and inconsistent of the three hazard species. These fluctuations cannot be explained by specific criteria such as migratory behaviour, seasonality, or hunting behaviour. Instead, the Straw-necked Ibis population appears to be strongly linked to food availability (leading to birds being attracted to the airport precincts, and increased breeding).*
Study Area		
R1.4	L 164-170 Authors postulate hypotheses on factors influencing the space use by Nankeen kestrel, but do not return to this point explicitly in the results and discussion section. Results and inferences pertaining to this hypothesis can be added in Discussion as	We thank the reviewer for this suggestion and have moved the discussion of Nankeen Kestrel hunting behaviour and its effect on the daily count to the Discussion section.

	an explanation for seasonal changes in the species' space use/numbers.	
Methods		
R1.5.1	Methods overlap with Results and need substantial revision to improve the flow and clarity.	The Methods and Results sections have been substantially revised to clearly separate Methods from Results.<br>The following text blocks have been moved from Results to Methods sections:<br>• L397-404<br>• L420-431<br>• L435-440<br>• L443-448<br>• L463-470<br>• L498-506
R1.5.2	The statistical analysis around the ABC approach can be improved by including objective examination of whether some 'days' have higher collision risk (see suggestion below in point 7).	We thank the reviewer for this suggestion. We have adopted the approach suggested by the reviewer in point 7 and applied GLM to associate characteristics of days to collision likelihood.
R1.5.3	Theoretical concepts behind the three methods – Algebraic modelling, Bayesian networks and K-means cluster analysis are explained well, but are sometimes superfluous as these techniques are widely used, and these sections can be reduced by referring to relevant literature wherever applicable, to keep the text tight, to the point, and focused on the application of the technique.	Much of the underpinning theoretical concepts have been reduced/removed in line with the reviewer's suggestion.
R1.5.4	More details on how these techniques have been applied such as variables and analytical treatments need to be mentioned explicitly, so that the approach can be clearly understood and replicated.	We have updated the Methods section to better indicate the variables and analytical treatments, including software and libraries, that were used in the study.
R1.5.6	Some analysis that these techniques depend on such as PCA, ROC, and validation techniques for Cluster analysis are not mentioned in Methods and appear abruptly in Results, making it difficult to follow how the methods were applied.	We have carefully reviewed the manuscript to address this issue. Sections of text in the Results section that introduce a method or technique (e.g., ROC, PCA, internal cluster validation such as silhouette maps) have now been moved to Methods, with only actual results reported in the Results section.
R1.6	L 234 – 320 Algebraic modelling approach has been explained in sufficient detail and clarity to allow replication. However, the content is verbose and can be revised for brevity. For instance, repetition of the mathematical notation in L274 – 276 is redundant, and L283-301 explanations of Gaussian and Sigmoid functions can be reduced.	We have removed the repeated mathematical notation in L274-L276 and reduced the text describing gaussian and sigmoid functions.
R1.7	L 304 – 309 The choice of expected abundance thresholds in classifying collision	We do thank the reviewer for this suggestion. We did use Binomial GLM to model strike probability on daily

	likelihoods as 'low', 'medium', 'high' is subjective and arbitrary. No statistical treatment has been presented that relates expected abundance to air strike probability. An objective way would be to predict collision probability using binomial GLM (logistic regression) by modelling air strike (1/0) on daily expected abundance of hazard species (eh), and to subsequently classify abundance thresholds based on stipulated cut-offs of predicted collision probability.	abundance and seasonality values. From this modelling we were able to determine cut-offs. As the modelling was binary (0/1), we changed the formulation of the collision likelihood function so that the function returns either 'Low' or 'Elevated' as values for collision likelihood.<br><br>Details of the GLM have been added to the Methods section.
R1.8	L 310 Bayesian networks have been used to examine causal relationships between factors leading to collisions. Which factors / variables have been examined in this study are not mentioned in Methods.	The variables used have been included in the Methods section (in Table 2).
R1.9	L 316 In Bayesian networks, a node (factor) has a conditional probability distribution table for each parent node (causal factors) that explains the causal relationships. However, these tables are missing from the Results. Only the networks have been shown in Results which tells readers that X leads to Y but not how X influences Y that is vital for this MS – to understand how seasonal environmental changes can lead to conditions promoting collisions. I return to this point in the Results.	There are 30 such conditional probability tables (one for each node of each Bayesian Network Model). To make the tables available to reviewers, and ultimately readers, we have created a github repository which we reference in the MS.<br><br>https://github.com/robertandrews59/ABCBirdstrike<br><br>**NB.** As we are proposing to release additional Brisbane Airport Corporation related data, we have approached BAC Risk and Governance to grant approval for the release. As approval has not yet been granted, the github repository is set as 'private'.
R1.10	L 317-333 Content is verbose and can be revised for brevity	Some of the content has been moved to the Discussion section and the content in the lines indicated by the reviewer have been revised for brevity.
R1.11	In (K-means) Clustering, L 338 Although the objective was 'to see whether particular day types are associated with bird strikes', the following section on cluster analysis does not explain how this objective is answered from the knowledge of 'similar days/conditions' groups.	We have added a sentence describing how we relate day types and clusters.<br><br>*Thus, in our approach, each cluster will represent a day 'type' and the principal components, identified by PCA, will give insights into the data attributes that differentiate between day types.*
R1.12	L 339-350 & 370-378 Given that cluster analysis is a very widely used technique, parts of the section can be reduced by citing relevant literature.	We have reduced the description of K-means clustering.
R1.13	L 351-359 Data standardization is a widely used technique and can be explained in a single sentence with relevant citation.	We have adopted the reviewer's recommendation and reduced data standardisation to a single sentence with references.
Results		
	Much of the Results are actually details of methods or interpretation of results that should be shifted to Methods and Discussion sections, respectively. Results should provide	We thank the reviewer for this observation. We have carefully reviewed the manuscript and moved material

	adequate reporting of the output statistics of each analysis (see detailed suggestions below).	not directly reporting results to either the Methods or Discussion section.<br><br>Our response to R1.5.1 details the blocks of text moved from Results to Methods.<br><br>The following lines have been moved to the Discussion section:<br>• L164-173<br>• L322-333<br>• L397-403
R1.14	Figures lack legends, axis labels and have been presented without diligence that make them difficult to fully comprehend. There are too many figures, and figures of similar type (Fig 1-3, Fig 4-6, Fig 8-10, Fig 11-12, Fig 13-15) can be grouped with species names as labels / icons. Other recommendations specific to each figure are as follows:	Fig 1-3 have been reworked to include y-axis on the left, x and y axis titles, and a legend has been included. These figures have been grouped with individual figures labelled with respective hazard species names.<br><br>Figures 4-6 have been replaced (in the Methods section) with figures that show Bayesian Network model configuration for each of the three hazard species. These figures have also been grouped with individual figures labelled with respective hazard species names.<br><br>Figures 8-10 have been reworked to include days per cluster values. These figures have also been grouped with individual figures labelled with respective hazard species names.<br><br>We have left Figures 11-12 as we are concerned that grouping the figures will reduce their size and make them unreadable.<br><br>Figures 13-15 have been grouped.
R1.15	Fig 1-3: Shift y-axis to the left and remove chart titles.	This has been done.
R1.16	Fig 4-6: These figures show what factors influence collision likelihood. However, the tables/graphs accompanying child nodes seem to be unconditional data distributions that do not allow readers to interpret the nature of the relationships between nodes. Authors can think of presenting the conditional probability tables of child nodes as contingency tables on edges/arrows, or some other concise, interpretable ways, so that readers (including managers) can understand how some environmental changes can promote factors that ultimately lead to higher collision risk.	We thank the reviewer for this suggestion. We have modified images of each of the three trained Bayesian Network models to include: (i) strength of influence indicators, which show the degree to which a parent node influences a child node – strength of influence is given as width of arc connecting the nodes, and (ii) subsets of the conditional probability table for Likelihood to show states of indicative variables for Likelihood (High) and Likelihood (Low).<br><br>To allow readers to understand how some environmental changes can affect collision likelihood, we have made the full set of conditional probability tables available on our github repository:-<br><br>https://github.com/robertandrews59/ABCBirdstrike
R1.17	L 381-389 should be removed and included in the figure legend	This has been done.

R1.18	L 390-392 & L 405 & L 415 Collision likelihood based on Algebraic Modelling need to be revised based on the suggestion in point 7.	We have added that the cut-offs were determined through application of GLM of (expected) abundance to strike probability (in line with suggestion in R1.7).
R1.19	L 397-403 should be included in Discussion and removed from here.	The indicated text has been moved to Discussion as suggested.
R1.20	L 420-430 details on Bayesian network analysis should be included in Methods and removed from here.	This has been done.
R1.21	L 438 – 448 description of ROC should be included in Methods and removed from here.	This has been done.
R1.22	L 450 – 462 details of Cluster analysis should be shifted from here to Methods	This has been done.
R1.23	L 457 is confusing, as it mentions that PCA can be used as a variable for Algebraic Modelling; however, this variable was not used in Algebraic Modelling and was not referred there. There are several such ambiguous, half-explained statements throughout the MS that makes the methods difficult to follow and replicate.	We have removed the reference to attributes identified in PCA being used in algebraic modelling.
R1.24	L 463 – 475 details of Cluster analysis validation should be shifted from here to Methods.	This has been done.
R1.25	L 496 Table 4 showing the number of strikes as a function of cluster type is non-informative as we do not know how many days are there in each cluster, to be able to infer if the frequency of strikes was more in one cluster than other. Also cluster difference in collision probability has not been tested statistically, hence the inferences are not reliable. Authors should revise this section (and the corresponding Methods) by including a statistical test of difference in collision probability between day clusters, similar to my suggestion in point 7.	The number of days included in each cluster have been added to table 4.<br><br>We have addressed this suggestion by calculating, for each hazard species, collision likelihood (based on observed strikes) over the entire dataset, and then comparing this with collision likelihood within clusters. The test of significance applied was "overlap" of confidence intervals (at 95% CI). If the confidence interval of a cluster overlapped with the confidence interval of the hazard species, the collision likelihoods were taken to be not significantly different. This has been added to the Methods section and reported in Results.
R1.26	L 497 – 511 details of Principal Component Analysis should be shifted from here to Methods. PCA methods should clearly list the variables included and analytical details (data standardization, correlation vs covariance matrix use etc.) in Methods. PCA results should include the variance explained and variable loadings of components in Results.	The details of the PCA have been shifted to Methods as suggested.<br><br>The detailed PCA results, including variable loadings, are included in the Supporting Information section.
R1.27	Cluster analysis results should include cluster centroid descriptions in terms of variable means for each hazard species and number of days in each cluster in Results so that	The number of observations in each cluster is provided in the Results section (in Table 4). For clarity, they have been included in the caption of each figure.

	readers (including managers) can understand how days are grouped based on conditions and which condition set increases the risk of collision.	
Discussion and Conclusion		
R1.28	Discussion is weak and largely reiterates the advantages of the approach developed in the MS. It can be strengthened by: **a)** discussing the findings of applying this approach to the current study in terms of which dynamic factors increased collision risk of a hazard species, and interpreting these results from ecological perspective (see comments in Results section); **b)** referring to literature on how bird strikes are mitigated across the world beyond simple categorization of hazard species by their risk/severity; and **c)** how the approach developed in the MS can help advance these current approaches of bird strike mitigation, thereby expanding the scope of the work.	The Discussion section has been revised in line with the reviewer's suggestions. Material from the Results section that was more interpretation of results than pure results, has been moved to the Discussion section. Literature relating to mitigation of bird strikes around the world has been included. We add to the manuscript a positioning of approach among existing mitigation approaches and point out the gap that our approach fills and thus, how it helps advance the state of the art.
R1.29	L 556 'PCA revealed that number of hazard species … were strong indicators of strikes' : It is not clear how authors jump to this inference, as Methods do not clarify how daily collision frequency was related to Principal Component(s), and Results do not report any such statistic. The PCA figures 13-15 only show loadings and relative contributions of variables on components, not which component influences collision risk and how. Again refer to my suggestion in point 7 and revise the analytical approach in the MS accordingly.	We have tempered this statement to better reflect PCA analysis as indicating attributes contributing to clustering. We observe that the attributes identified through PCA are those used in GLM modelling described in Methods and Results for algebraic modelling.
R1.30	Conclusion reiterates the approaches used, much of which have already been covered. Instead, it can be reduced to a few important concluding statements on the application of these techniques in reducing bird strike problems.	We thank the reviewer for this suggestion. The Conclusion has been rewritten to emphasise the contributions of the techniques to bird strike, and how the models may be employed to reduce risk of collision.

**Comment #**	**Comment**	**Author Team Response**
**Reviewer 2 Comments**		
R2.0.1	The paper presents three ways to inform collision risk from wild birds. This is an important study that has real world applications. The authors make a case for their study by referencing other risk assessment frameworks and approaches.	We thank the reviewer for this observation. We have significantly revamped the Methods to make clear our approaches, assumptions, and tools used in conducting the study.

	However being a study that describes novel methods for risk assessment, a solid, readable and fluid methods section is indispensable to the manuscript, which is currently lacking.	
R2.0.2	There is scope for clarifying description of methods. In particular, the descriptions are presently difficult to follow as the important terms used in the calculations are not ordered. One has to refer to previous pages to understand.	In accordance with the reviewer's comments, we have significantly revised the manuscript and moved material out of Results and into Methods section to make each section more self-contained and readable. In particular, the following blocks of text have been moved from Results to Methods:<br>• L397-404<br>• L420-431<br>• L435-440<br>• L443-448<br>• L463-470<br>L498-506
R2.0.3	The conceptual parts of the 3 methods could be included as they apply to the problem being presented, or skipped altogether.	We have addressed this in Methods.
R2.0.4	Methods and results sections are intermixed in many ways.	See response to R2.02. above.
R2.0.5	The Introduction and Discussion are relatively well drafted, however the same clarity does not exist in the Methods and Results sections.	See response to R2.0.2 above.
R2.0.6	An accuracy test of the three methods, using predicted collision risk and actual data of collision could be a valuable addition.	Accuracy of Algebraic and Bayesian Network models have been included in Results. Accuracy of Clustering is also shown in Results through showing the distribution of strikes across clusters.
R2.0.7	Authors may check for the correct use of wildlife ecology terminology viz., population, species, abundance etc. 'Populations' are mostly estimates, and not absolute numbers.	We thank the reviewer for pointing this out. We have used 'count' rather than population throughout the manuscript.
R2.0.8	Moreover non-detection of birds has not been included in the methods, while it has been acknowledged in results (lines 402-403).	We have added a sentence about non-detection and its affect on count, proximity count, and abundance in Methods.<br>*We note that (i) non-detection of individuals from the hazard species (either through not being observed in one, or any zone during the daily count, or not being involved in any harassment activities on a given day) will result in* $count_z^h$ *being 0 for each such zone on the airfield, and will be reflected in the* $count_p^h$ *and* $abundance_h$ *values for the day, and (ii) overall wildlife abundance on the airfield may be calculated similarly as for a single species.*

R2.1	Not clear if how variation in bird numbers in Paton's approach is taken into account, and how this approach does not account for seasonal/environmental changes. Authors may elaborate on this.	The issue with Paton's approach is that it provides a means of ranking hazard species according to risk, but does not provide a means of deriving a risk assessment on any given day.
R2.2	WHM?	Wildlife Hazard Management. This is introduced in the second sentence of the Introduction.
R2.3	Study hazard species are those that have a history of collision with aircrafts. However the authors have stated in the introduction that it is important to include species that may not have a history but may still be potential hazards	Two of the species, Nankeen Kestrel and Cattle Egret, have a history of collision. Straw-necked Ibis is a species that is abundant on the airfield, frequently observed in the vicinity of runways and taxiways, but which is rarely involved in strikes.<br><br>We have tried to clarify this in the Materials and Methods section where we describe the hazard species.
R2.4	For context, it may be useful to define the main food source (for reference in line 225), habitat requirements/nesting ecology of the hazard species.	Food sources and breeding have been added to the description of the hazard species in the Study Area and Data Collection section.
R2.5	Para beginning at line 237: for clarity, I suggest the authors provide some context of the references provided here (eg. Carter in his study on risk assessment and prioritisation of wildlife hazards, the author used ….).	We have amended this paragraph in line with the reviewer's suggestion as:<br><br>*Paton, when describing his risk assessment model [15], outlined various likelihood indicators including (i) relative abundance, (ii) frequency of occurrence and or area of occurrence. Carter, in his study on risk assessment and prioritisation of wildlife hazards [11] the author includes (i) overall population…*
R2.6	Line 243 – 245: does this clustering refer to the k-means clustering? The meaning is unclear. Also a reference is probably missing at the end of the sentence.	Yes. This does refer to our clustering results. We thank the reviewer for pointing out the missing internal reference. This has been amended.
R2.7	Line 347: number of hazard species should be written as 'number of individuals belonging to hazard species'.	Thank you. This has been amended as suggested.
R2.8	Lines 267 and beyond: the term 'population' doesn't seem appropriate here. Authors could use 'count per zone' or 'individuals per zone'. Note that 'sum of hazard species .. ' and number of individuals of the species (I think the authors mean this) are two separate things.	Thank you. We have changed "population" to "count".
R2.9	Lines 266 – 271: the language of this explanation is not clear.	This section has been rewritten and now includes the relevant equations directly following the textual descriptions of each of the three measures (count, proximity count, and abundance).
R2.10	Equation after line 274: q has not been defined. Do the authors mean p?	No. p refers to a single proximity area. The variable q is used to represent summing over all proximity areas.
R2.11	Line 353-359: standardisation is a standard practice, authors need not explain this.	Thank you. We have removed the explanation.

R2.12	Line 361 What was done in references 23 and 24 need to be defined here. What is the average 0.1 value, and how is the 0.5 value set as threshold?	This section has been rewritten in the paragraph that explains the use of the R package and functions.
R2.13	Lines 364 – 366: describe how.	This section has been rewritten in the paragraph that explains the use of the R package and functions.
R2.14	Lines 367 – 369: use this to substantiate on what analyses were done using these packages in R, and which functions were used for which purpose (instead of not defining how average values and recommended number of clusters were derived – ref to comments above).	This paragraph has been rewritten to indicate which R functions were used to perform which test.<br>*The computational environment was the R statistical modelling software version 3.6.3 [29]. The get clust tendency() function was used to determine the Hopkins statistic [26] as an indicator of suitability of the data for clustering. The returned (average) value of 0.1, being close to zero, indicated the data was suitable for clustering. The fviz nbclust() function, using each of the three methods provided for estimating the optimal number of clusters, gave recommended number of clusters, for each species, as two, i.e. K=2. The kmeans() function was used as the implementation of K-means clustering. The clvalid() function was used to return internal and external measures of validity. The PCA() and get pca var() functions were used to return principal components.*
R2.15	Lines 420 – 429: methodological details which are incorrectly placed in the results section.	Thank you. This text has been moved to the Methods section.
