# Supplementary material for: Three novel bird strike likelihood modelling techniques: The case of Brisbane Airport, Australia
Source: PLoS One. 2022 Dec 8;17(12):e0277794. doi: 10.1371/journal.pone.0277794 (PMC9731475; doi:10.1371/journal.pone.0277794)
Supplement: Attachment — Submitted filename: 20221022 Response to Reviewers Letter.docx [file pone.0277794.s006.docx]

**Response to Reviewers**

**Due:** Oct 22, 2022

**Ref:** PONE-D-21-25171 “The ABC of Bird Strikes at BAC: Modelling bird strike likelihood at Brisbane Airport”.

**Revised Title:** Three Novel Bird Strike Likelihood Modelling Techniques: The Case of Brisbane Airport, Australia

| **Comment #** | **Comment** | **Author Team Response** |
| --- | --- | --- |
| **Editor’s Comments** | | |
| E.1 | I suggest that you take into consideration the recommendations of both reviewers. In particular reviewer 3 brings two very important issues. The first is the differentiation between "likelihood" and "risk", you should specify what you performed (in my opinion you did likelihood, but not risk). If so, correct in the title and in some parts of the text.  Another option is to detail in methods that likelihood was used as a proxy for risk, but it should be adequately justified with references and explaining how you account for severity. | We have ensured in the title, and relevant parts of the text, that we make it clear we deal only with likelihood of bird strike, rather than risk (as a combination of likelihood and severity). |
| E.2 | Another critical point indicated by Reviewer 3 is that at no point is it evaluated which of the 3 techniques is better, or if it is the combination of them. I consider that including some of this could greatly improve the conclusion of the paper. | The three techniques are not meant to be in competition with each other - a comparison would give that false impression - rather they are complementary approaches. Hence, they should be used in conjunction with each other as they have different strengths and weaknesses. If one of the approaches predicts a likely strike, action should already be taken. This has been made clearer in the paper. We have added, in the Discussion section:  *As for practical application of the ABC approach to collision likelihood, we feel that the techniques should be applied in parallel, and if any one of the techniques predicts a heightened likelihood of collision, actions should be taken. AS discussed earlier, the techniques give indications of the factors leading to heightened collision likelihood. These could be used to inform mitigation actions.* |
| E.3 | Moreover, I include two additional recommendations. The title is uninformative and could even be misleading due to the presence of so many abbreviations. I understand that a play on words may have been created, but for non-English speaking audiences it is not clear.  I suggest avoiding the inclusion of abbreviations (e.g. ABC or BAC) or local names without their country. A suggestion may be “Evaluation of risk assessment techniques to model bird strikes: the case of Brisbane Airport in Australia”.  Authors should include the meaning of the abbreviation ABC in the abstract (e.g. in line 39). | We have retitled the paper as:  *Three novel bird strike likelihood modelling techniques: The case of Brisbane Airport, Australia*  This change takes into account both the Editor’s suggestions (no acronyms, include country), and Reviewer 3’s concerns about being clear that we modelled ‘likelihood’ rather than ‘risk’.  We have amended the abstract to (i) make it clear the paper addresses likelihood, rather than risk. We have also made the meaning of the ABC acronym clear. The revised section of the Abstract is  *In this article we focus on likelihood of bird strike and introduce three distinct, but complementary, assessment techniques, i.e., Algebraic, Bayesian, and Clustering (ABC) for measuring the likelihood of bird strike in the face of constantly changing environmental conditions.* |

| **Comment #** | **Comment** | **Author Team Response** |
| --- | --- | --- |
| **Reviewer 2 Comments** | | |
| General Comments | | |
| R2.1 | The manuscript is considerably improved, and the authors have addressed all previous comments/suggestions. I recommend few additional suggestions for enhancing the readability and understanding of the general idea of the paper. I would also like to commend the authors on carrying out this important study. | Thank you. |
| R2.2 | Authors could add a mention of how frequently would the models be updated with emerging information (recent collision information, local weather changes, novel hazard species)? | This is a good point. We have made mention, in the Conclusion, of this as a topic of future research as the sensitivity of the models to emerging information is something that we did not investigate. We have also added, as future research, an investigation into the optimum ‘window’ of historical data that should be used to give the best modelling results.  *Further, the variables used by the models are those routinely collected by airport Wildlife Hazard Management teams thus implementing the techniques at other airports should not add any burden to data collection, or require changes in data collection practices. Areas for future research around data include (i) how frequently should the historical environmental and collision data be updated in order to keep the models current, i.e., reflective of the actual collision likelihood at the airport, and (ii) what `window' of historical data is optimal for modelling.* |
| R2.3 | Curious why all three methods were carried out in different software, and not one. Authors could add a line justifying this. | We have included the following in the Conclusion to address this point:  *Lastly, this work was developmental. As such, software used was chosen that directly supported the models, and provided appropriate analysis and visualisation capabilities. Future development may include development of an integrated platform making all the techniques available in the one tool.* |
| R2.4 | Figure and table titles require editing (typos) and work (addition of information to make it comprehensive by itself, and not dependent on text). | This has been done. Figures 1, 2, 4, 5, 6, 7, 11 have been modified as has the caption for Tables 3 and 4. |
| Abstract | | |
| R2.5 | Line 27: and rare bird extinction Line 29: and bird populations. | We have amended the Abstract as requested.  We also adjusted the related sentences in the Introduction. |
| Introduction | | |
| R2.6 | Line 161: raptors (“preying on?” small mammals and insects) | This has been revised as:  *small, non-migratory, raptors (preying on small mammals and insects)* |
| R2.7 | Line 177-179: is this a repetition of line 150-153?. | There is indeed some duplication here. Thank you for catching this. Lines 177-179 have been removed. The reference to Table 6 – strike details, has been moved up to line 153. |
| Methods | | |
| R2.8 | Line 232: “includes ten likelihood includes ten indicators including” – not clear | Thank you for pointing this out. This has been rectified as:  *includes ten likelihood indicators including …* |
| R2.9 | Table 2, page 13: Type column for Zone’s population and All population: continuous (instead of continues) | This has been rectified. |
| R2.10 | Fig 1, page 14: typos in title (Straw-necked ibis – at two places) | Thank you. This has been rectified. |
| R2.11 | Line 336: to demonstrates | Changed to:  *that demonstrates* |
| R2.12 | Line 390: Connect explanation of silhouette coefficient and Dunn index with subsequent lines (392-395), in terms of which index/coefficient is a measure of what. Moreover, these terms do not appear in the results. | This has been addressed as:  *Internal cluster validation measures reflect on the compactness, connectedness, and the separation of the cluster partitions, and can be determined by the silhouette coefficient (with range [-1..1] [25]) and the Dunn index (with range [0..∞) [29]). The goal is to maximise both the silhouette coefficient and the Dunn index [30]. If the data set contains compact and well-separated clusters, the diameter of the clusters is expected to be small (silhouette coefficient will be close to 1 and the Dunn index will be large).* |
| Results | | |
| R2.13 | Table 3: Title above table, title needs to be more explanatory (instead of explanation in text) | Table caption has been modified as:  *Logistic Regression modelling results for each of the three hazard species showing model quality (accuracy and F Score), attribute with highest feature importance in the model, and threshold value that maximises strike likelihood prediction accuracy.* |
| R2.14 | Line 422: not clear why the split was necessary | We have added the following by way of explanation:  *Note that the general shape of the expected abundance curve for the Nankeen Kestrel is similar to that of the Cattle Egret, i.e, shows distinct seasonality. Unlike Cattle Egrets, Nankeen Kestrels are involved in collisions throughout the year. We found we got better predictive accuracy by splitting the observational data into two distinct sets at the change in seasonality.* |
| R2.15 | Line 429-432: two sentences with repetitive meaning | Thank you. This has been rectified. |
| R2.16 | Line 434: an explanation of one the figures would add value to this section. | We have added the following by way of explaining these figures:  *For instance, in Fig 4 (i) Season strongly influences Seasonal Behaviour (the Cattle Egret is a migratory species and breeds in summer), and (ii) the likelihood of collision is high when the number of Cattle Egrets Observed is high, with highest collision likelihood when the Population per Zone is High, the number of Cattle Egrets Observed is High, the overall Population  of hazard species is Medium, and the number of Aircraft Movements is High.* |
| R2.17 | k-means clustering; lines 443-453: repetition of methods. Authors could add a few lines “…we found that….” Lines 454-456: text better suited/more appropriate for figure title | We thank the reviewer for this observation. These lines have been removed from Results section. We have added:  *Clustering results are visualised in the 2-dimensional plots in Fig 7 with cluster validity measures shown in Table 4.* |
| R2.18 | Lines 454-456: text better suited/more appropriate for figure title | We have moved this text to the caption of the figure as suggested. |
| R2.19 | Table 4: title needs revision; cluster tendency is a new term not been mentioned before (in methods) | We have replaced this with Silhouette Score as described in the Methods section. We have also revised the table caption. |
| R2.20 | Figures 8 and 9: reason for placing figures for species as such (1:2)? Figure titles need to explain coefficients etc mentioned in the plot. | The small images in Figure 9 have been separated into two images and made the same size as Figure 8. This was purely space saving in the original form. The captions have been changed to be more informative, i.e., explain the coefficients mentioned in the plot. |
| R2.21 | Line 460: is average silhouette score the same as silhouette coefficient (mentioned in line 390)? Authors should use the same term throughout the manuscript. | Silhouette score and coefficient are interchangeable. We have adopted “coefficient” throughout the manuscript. |
| Discussion | | |
| R2.22 | Line 568: they are rarely in close to proximity | Thanks. This has been rectified (superfluous “to” removed). |
| R2.23 | Line 572: when overlaid with strike information, provides | Changed to:  *When overlaid with strike information, expected abundance provides…* |
| R2.24 | Line 600: days into clusters (?) | Changed to:  *clustering generated a set of prototypical ‘day types’* |

| **Comment #** | **Comment** | **Author Team Response** |
| --- | --- | --- |
| **Reviewer 3 Comments** | | |
| General Comments | | |
| R3.1 | Risk assessment procedures, as crucial as they are, do have their limitations in practice as identified by the authors. Hence, I appreciate their approach of investigating new methods to improve the data availability for wildlife controllers in the field. | Thank you. |
| R3.2 | The authors clearly state in the text that they focus on likelihood, excluding the severity aspect. This is perfectly fine, however, by doing so, they do not create a risk but a likelihood model. This is indicated in the title, but throughout the text, there is still some mentioning of their models as “risk assessment models”. Please correct. | This has been corrected throughout. |
| R3.3 | It is interesting to see the three presented approaches as well as their results. What I am missing in the overall discussion is, how they can best be used in practice Lines 43-44 in the abstract state that each of the techniques meet the requirements – so would one be enough? Which one best? Or would a combination be wise after all? | The three techniques are not meant to be in competition with each other - a comparison would give that false impression - rather they are complementary approaches. Hence, they should be used in conjunction with each other as they have different strengths and weaknesses. If one of the approaches predicts a likely strike, action should already be taken. This has been made clearer in the paper. We have added, in the Discussion section:  *As for practical application of the ABC approach to collision likelihood, we feel that the techniques should be applied in parallel, and if any one of the techniques predicts a heightened likelihood of collision, actions should be taken. AS discussed earlier, the techniques give indications of the factors leading to heightened collision likelihood. These could be used to inform mitigation actions.* |
| R3.4 | In the conclusion you mention that the ABC approach is useful etc. But what exactly is the approach? Looking at each of the individual models? Combining their outcome? I am missing the big picture here, please enlighten me and the other readers in the discussion or the conclusions section. | Addressed in R3.3 above. |
| R3.5 | In addition, since the authors seem to strive to actually support risk assessment procedures in practice, I am missing an outlook on where they will take the models from here to actually get them to the airports and make them useable for the wildlife control teams. | We have made brief mention, in the Conclusion, of the steps necessary to realise the ABC approach in a form useful to airports.  *Diffusion theory [] is concerned with the uptake and spread of innovations. It tells us, among others, that technology adoption is driven by not only technological factors, but adoption pathways, time, and the social system around the innovation. In this context, Realisation of the ABC approach as a tool for use in practice has both technological and awareness/acceptance considerations. The technological side will require, among other things, the implementation of each technique in an integrated platform, thus making all the techniques available in the one tool. Such a tool would also require options to allow wildlife control teams to configure routinely collected data for presentation to the models, and to define hazard species relevant to the airport at which the approach was to be deployed.*  *Awareness will require disseminating information about the approach to the industry through for instance wildlife hazard working groups. Acceptance will require a quantum of early adopter airports and their subsequent communication of positive feedback to other airports, leading to more widespread adoption and ultimately approval or even enforcement by from regulatory bodies.* |
| Comments on the Text | | |
| R3.6 | Source 9 (US Wildlife strikes 1990-2015) is outdated. The current version is 1990-2021, please update | Thank you for this information. The reference, and related number of people killed and aircraft destroyed have also been updated in the manuscript. |
| R3.7 | There is a very hard content-related break between the intro and the methods section (l 112-118). Help the reader by ending the introduction /starting the introduction with a brief reiteration of what you are going to present next. E.g. “to develop these models, a case study was performed at Brisbane airport”. | Thank you for this suggestion. We have added the suggested text to the beginning of the Material and Methods section. |
| R3.8 | L 138: the text indicates that you selected three out of multiple hazardous species. Is this the case or are these THE three (most) hazardous species? In case it was three out of multiple, please state why you selected those. If it was the three, please clarify. | The set of hazard species investigated in this study was nominated by the collaborating airport. Brisbane airport’s Wildlife Hazard Management team indicated the three species included in the study were of most interest to the team because (i) Cattle Egrets and Nankeen Kestrels are among the species most involved in collisions (the most frequently struck species are Flying Foxes and other species of bats for which little or no observational data was available due to these species being nocturnal), and (ii) Straw-necked Ibis occur in very large numbers (flocks of thousands of individuals) on the airport.  We have clarified this as:  *BAC nominated the three hazard species it considered as being high risk species for inclusion in the study. The Cattle Egret (Bubulcus ibis) and Nankeen Kestrel (Falco cenchroides) were included due to their comparatively frequent involvement in collisions. The Straw-necked Ibis (Threskiornis spinicollis) was included due to the large numbers of this species (flocks of thousands of individuals) frequently observed on the airfield.* |
| R3.9 | L 182-183: superfluous, starting the methods with what is written in l 184 is perfectly sufficient. | We have removed these lines as suggested. |
| R3.10 | Table 1: What about limitations? Are these hidden in the requirements? If so, I would relabel the “requirements” descriptor. If not, limitations should be added for the sake of completeness. | We have added a separate row to the table indicating limitations of each method. |
| R3.11 | L 279: I am missing a source here as well as equation numbers on this page. | We have added sources as:  *In [20] and [21] techniques are described for constructive function approximation using Gaussian kernels and sigmoid functions respectively.*  20. Hahm NW, Hong BI. Constructive approximation by Gaussian neural networks. Honam Mathematical Journal. 2012;34(3):341–349.  21. Costarelli D, Spigler R, et al. Constructive approximation by superposition of sigmoidal functions. Analysis in Theory and Applications. 2013;29(2):169–196.  Equation numbers have also been added. |
| R3.12 | Gaußian and Sigmoid functions come out of the blue. Please briefly state what they are and why they are relevant after l 279. | This has been addressed in response to R.11. |
| R3.13 | L 315-317: This is a strong statement. Either you have to provide a source or some reasoning why you believe this to back it. | We have changed this paragraph as:  *Bayesian approaches have been mentioned in relation to climate and environmental factors influencing forest management [24], for imputing missing values in a data set used for analysis of engine failure following bird strike [25], and for estimating structural damage to aircraft following a bird strike [26]. To date, however, Bayesian Networks have not been applied to assess factors influencing the likelihood of wildlife hazard strike occurrence at airports. To address this gap, we show how Bayesian Networks can be utilised in the context of bird strike likelihood.* |
| R3.14 | Equation before Table 2 – how can a likelihood be a function of a risk level? This does not make sense at first sight. Please elaborate and give an explanation what the risk level includes and how it is measured. | Thank you for pointing this out. This is actually intended to be a weighting based on the distance from the runway. Rather than calling it a “Location Risk”, we have changed this to be called a “Proximity Weight”. This is in line with the proximity weighting described in the Algebraic Modelling section. |
| R3.15 | Figures in general (except F8): Text of labels / in bubbles is extremely small and thus almost illegible. Please increase the font sizes. You might want to change color-coding to different shades of grey for readers who print the paper in grey scale (such as myself). | We have done our best to increase the font sizes in each figure, and to edit the layout of graph nodes to make better use of space. This has allowed us to make the figures themselves somewhat larger. This relates to Figures 1, 2, 4, 5, 6, 7, 11 |
| R3.16 | L 442: please make this a full sentence. | Reviewer 2 pointed out that this block of text was largely a repetition of what was described in the Methods. In response to Reviewer 2’s observation, we removed the repeated text from Results.  The revised text, Methods, now reads:  *Accordingly, we took the following steps.*   1. *Applied ….* |
| R3.17 | 514-524: Would this information not be more suitable in the intro? And please add some sources for the individual systems/models. Even though in the business for quite a while, I have never heard of the Swiss/Dutch Bird Migration Model. Is this really its name? Please add a reference. | We have moved this paragraph to the Introduction as suggested. Sources/references have been added for each system.  *Various wildlife hazard risk assessment approaches have been proposed/implemented around the world. Common to all, is the aim of reducing the likelihood of birdstrike, however differences in approaches characterised by their (i) sphere of application (local airport to (inter-)national), (ii) forecast horizon (static to real-time), and (iii) realisation (as being technique/algorithm, a model, or an implemented system). Here we take the definitions of technique (theoretical, mathematically based structure), model (framework of techniques and algorithms to describe/predict real word situation), and system (interacting components including models, sensors, communications networks to utlise model outputs) from [13]. The following models/systems are at large spatial scale, i.e., larger scale than individual airport. The models/systems differ also in their forecast horizon. For example, real-time birdstrike warning systems include the Avian Hazard Advisory System (AHAS) [14] in the United States, the Dutch Radar Observation of Bird Intensities system (Dutch ROBIN system - https://www.robinradar.com/) in the Netherlands. In Europe, the FlySafe Bird Avoidance Model [15] provides near real-time information and forecast on large scale bird movement in the air space of The Netherlands, Germany and Belgium. Longer forecast horizon model/systems include the United States and North America Bird Avoidance Model (USBAM) [16], the German birdstrike risk forecast model [13], and the Swiss/Dutch Dynamic Bird Migration Model [13, 17]. The USBAM is a statistical model, while the German birdstrike risk forecast model is a conceptual model, and the Swiss/Dutch dynamic bird migration model is a simulation model. Purely static, wildlife species risk ranking techniques/algorithms include Allan’s formula [11], Paton’s approach [18], and Carter’s formula [19] These techniques are intended for application at individual airports.*  NB. The Swiss/Dutch Model is not implemented in practice but is well described in [17].  [17] Nussbaumer R, Bauer S, Benoit L, Mariethoz G, Liechti F, Schmid B. Quantifying year-round nocturnal bird migration with a fluid dynamics model. Journal of the Royal Society Interface. 2021;18(179):20210194 |
| R3.18 | 532-540: This is interesting but in large parts a repetition of the results – please make that paragraph more concise. | Thanks for this observation. The paragraph has been revised as:  *A strength of the Algebraic approach is that it clearly visualised the seasonal nature of the abundance of the hazard species included in this study. In particular, the migratory behaviour of the Cattle Egret is evident as is the hunting behaviour of Nankeen Kestrels across the year. For the Cattle Egret, peak abundance corresponds with the period where the most strikes involving Cattle Egrets were observed. For the Nankeen Kestrel we hypothesise that the seasonal abundance is related to the bird’s hunting behaviour and the fixed time of the day at which the daily count is conducted, rather than actual changes in the number of birds on the airfield.* |
| R3.19 | I realize that you discuss the results of the models in different level of details. Why is this? I would recommend to revisit this section and investigate the options to bring more balance into it. In addition, I would expect some mapping to literature and/or observations from BAC or other airports. Do the models reflect reality well? | We have considered the results and discussion and have added clarifying sentences in various places to try to bring more balance to the discussion.  We have included in the Introduction a short paragraph dealing with recent literature involving techniques same/similar to those described in this article.  *Little attention has been paid to applying machine learning to aspects of birdstrike risk. Rosa et al. [20] applied 6 different machine learning techniques to data collected by marine radar in Portugal. This study concluded that all techniques were able to distinguish between birds and stationary objects, but distinguishing between different species of birds was less successful. Recently, Nimmagadda et al. [21] applied decision tree and naive Bayesian approaches to the problem of predicting whether an airline crash has occurred due to a birdstrike. Verma et al. [22] provides a literature review of various techniques for prediction of general aviation accidents including, among others, Bayesian Networks, Artificial Neural Networks, data mining, and ensemble approaches. None of these techniques were specific to predicting likelihood of wildlife collision on/near an airfield.* |
| R3.20 | L 558-559: the bird’s weren’t active when? On the day when the considered strike occurred? Please clarify. | This refers to days where a strike involving a Nankeen Kestrel was recorded yet no Nankeen Kestrels were observed/counted during the daily count.  This has been changed to:  *Expert opinion from the BAC WHM indicated that the population of Nankeen Kestrels on the airfield remained constant throughout the year, yet the abundance figures (derived from the daily count) show fewer birds counted during the warmer period of the year, with more strikes occurring in this period. We ascribe this to the timing of the daily count and the different hunting behaviours exhibited by Nankeen  Kestrels at different times of the year. That is, the birds were on/around the airport****, but weren’t actively hunting or flying at the time the daily count was conducted, i.e., they were perching out of sight and hence were not observed during the daily count****. A point worth noting is that, for 7 of the 20 strike events involving Nankeen Kestrels recorded during the study period, there were no Nankeen Kestrels recorded in the daily count or included in harassment activities conducted by the Wildlife Hazard Management team.* |
| R3.21 | L 572 it provides (‘it’ is missing). | This has been changed to:  *When overlaid with strike information, it provides an easy way to derive rules that quantify collision likelihood.* |
